# Ultrasensitive fluorescent aptasensor for MUC1 detection based on deoxyribonuclease I-aided target recycling signal amplification†

**DOI:** 10.1039/c8ra06498a

**Published:** 2018-09-14

**Authors:** Jun Zhang, Fengying Ran, Wenbo Zhou, Bing Shang, Fei Yu, Lun Wu, Wanbao Hu, Xueqin He, Qinhua Chen

**Affiliations:** Affiliated Dongfeng Hospital, Hubei University of Medicine Shiyan Hubei 442008 China cqh77@163.com +86 0719-8272283; Sinopharm Dongfeng Huaguo Hospital Shiyan 442008 Hubei China

## Abstract

A novel sensing strategy for sensitive detection of mucin 1 protein (MUC1) based on deoxyribonuclease I-aided target recycling signal amplification was proposed. In this paper, in the absence of MUC1, the MUC1 aptamer is absorbed on the surface of graphene oxide (GO) *via* π-stacking interactions. This results in quenching of the fluorescent label and no fluorescence signal is observed. Upon adding MUC1, the probe sequences could be specifically recognized by MUC1, leading to an increase in the fluorescence intensity. The detection limit is as low as 10 pg mL^−1^, and a linear range from 50 pg mL^−1^ to 100 ng mL^−1^. The assay is specific and sensitive, and successfully applied to the determination of MUC1 in spiked human serum, urine and saliva. Importantly, the proposed aptasensing strategy has great potential in detecting various protein and even cancer cells.

## Abstract 2

A novel sensing strategy for sensitive detection of mucin 1 protein (MUC1) based on deoxyribonuclease I-aided target recycling signal amplification was proposed.

## Graphical abstract

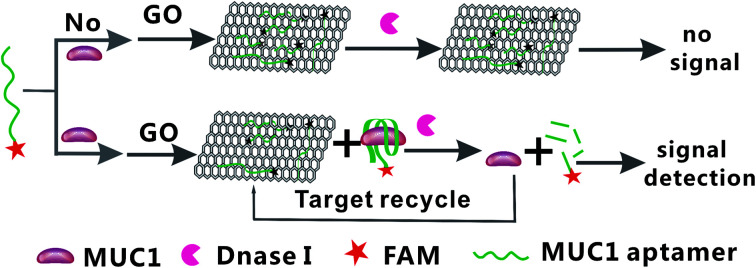

## 1. Introduction

At present, cancer is one of the most life-threatening diseases in the worldwide.^1^ The latest WHO figures show that 8.8 million people worldwide die from cancer every year, accounting for nearly 1/6 of deaths a year, and the figure is expected to increase to more than 21 million by 2030. Thus, it is important to develope effective methods for cancer therapy. Among them, the early identification and quantitative analysis of tumor biomarkers could provide a simpler and more effective method for monitoring tumor progression. It is of particular importance in early clinical diagnosis and treatment of cancer.

The mucin1 protein (MUC1), is a cell-surface associated glycoprotein of the mucin family,^2^ which contains a hydrophobic membrane-spanning domain of 31 amino acids, a cytoplasmic domain of 69 amino acids, and an extracellular domain consisting of a region of nearly identical repeats of 20 amino acids per repeat.^3,4^ MUC1 presents in a variety of malignant tumors and is over-expressed in almost all human epithelial, including breast, gastric, colorectal, lung, prostate, ovarian, pancreatic and bladder carcinomas;^5^ but, low levels or no expression in normal healthy tissues in normal cells. It is considered as a potential cancer biomarker. Thus, an sensitive detection of MUC1 is great importance in preliminary diagnosis of cancer. To date, several techniques including ELISA,^6^ surface plasmon resonance spectroscopy (SPR),^7^ colorimetric,^8^ electrochemistry,^9–11^ photoelectrochemical,^12^ electrochemical biosensors,^13–16^ electrochemiluminescence^17–19^ and fluorescence biosensors^20–23^ have been applied in the detection of MUC1. Among them, fluorescence assay is a well-known detection method with acknowledged advantages such as high sensitivity, easy readout, simplicity and the feasibility of quantification.^22,24^ Despite many advances in this field, these strategies still have shortcomings including the costly modification of fluorophores and potential false positive signals. Moreover, the sensitivity needs to be further improved. Therefore, it's important to develop an efficient and highly sensitive fluorescent strategy to detect MUC1 in the fundamental research and practical applications.

In recent years, there have some reports of mucin 1 glycoprotein based on MUC-1 aptamer. Aptamers are nucleic acid ligands (DNA or RNA) that can specifically bind protein, which are artificially synthesized and selected *in vitro* through exponential enrichment (SELEX).^25,26^ In comparison with other targeting agents, aptamers possess unique advantages such as small size, design flexibility, stability in harsh biological environments, easy for chemical modification as well as high specificity and high purity.^27,28^ These special characteristics will enable them be widely used biomarker discovery and biosensors.^29,30^ To date, a series of aptamer-based biosensors have been applied to detect VEGF_165_,^31^ EpCAM,^32^*Salmonella* paratyphi A,^33^ cocaine^34^ and so on. There have also been reported for detecting MUC1 using electrochemical,^35^ photoelectrochemical,^12^ colorimetric^8^ and fluorescent.^22^ But, among them, no standard method has been established. Therefore, a novel, highly sensitive and selective approaches for MUC1 detection need to be designed.

Graphene oxide (GO), a one-atom-thick two-dimensional carbon nanomaterial with a honeycomb structure. It's owing to unique features such as high mechanic strength, good water dispersibility and facile surface modification.^36,37^ GO has a high specific surface are a for binding plenty of molecules, such as nucleic acids, proteins, liposomes and pharmaceutical molecules.^38–40^ Moreover, GO is capable of quenching fluorescence with high efficiency, and can be used to construct GO-based sensors based on the principle of fluorescence resonance energy transfer (FRET).^41^ More importantly, GO can improve their stability in the biological environments, due to its protection capacity for biomolecules binded to its surface from enzymatic cleavage.^42^ Consequently, GO, as a superb nanomaterial, would be easily used in biosensors for biochemical analysis. On the other hand, to overcome these limitations, signal amplification strategies, enzyme-aided signal amplification have recently been developed to achieve the sensitive detection of biomolecules, including endonuclease, deoxyribonuclease I (DNase I), exdonuclease and DNA polymerase.^43^ Among them, DNase I, a common endonuclease, can digest single- and double-stranded DNA (dsDNA) into oligo- and mononucleotides. DNase I can cleave phosphodiester bonds to product many polynucleotides with 5′-phosphate and 3′-OH groups, but cannot digest RNA sequences. Thus, these makes it suitable for RNA analysis based on DNase I-assisted target recycling and signal amplification.^44^ Based on these findings, He *et al.*, described a approach nanographite-based fluorescent biosensor and DNase I for the amplified detection of *Salmonella enteritidis*,^43^ and Yan *et al.*, described a highly sensitive fluorescent aptasensor for *Salmonella* paratyphi A *via* DNase I-mediated cyclic signal amplification^33^ and so on. DNase I-aided signal amplification has the advantages of sensitive, reliable and specific. To meet the demands of the specificity and sensitivity of MUC1 detection, the development of an DNase I-aided signal amplification strategy is extremely urgent.

Hence, in this work, a very sensitive and specific aptasensor was designed to detect MUC1 based on DNase I-aided target recycling signal amplification. The MUC1 aptamer 5′ terminal was labeled with an FAM that amplifies fluorescence signals by cleaving the aptamer randomly. In the absence of MUC1, the MUC1 aptamer is strongly adsorbed onto GO, results in quenching of the fluorescent label and no fluorescence signal. Upon adding MUC1, it can recognize aptamer specifically and form aptamer/MUC1 complexes, resulting in the aptamer being away from GO. And then, when DNase I is introduced, enzymatic digestion reaction that releases the MUC1 for the next round occurs, leads to the accumulation a large amount of FAM fluorophores. The quantities of MUC1 can be achieved by fluorescence increment. Thus, a novel, sensitive, and specific aptasensor is obtained for assaying MUC1. Furthermore, it's successfully applied for detection of MUC1 in various spiked biological samples. One may believe that this sensing system possesses great potential for the monitoring of proteins, clinical diagnosis, and treatment.

## 2. Experimental section

### 2.1 Reagents and materials

The MUC1, EpCAM, BSA, PSA and VEGF were purchased from Cusabio Biotech Co. Ltd. (http://www.cusabio.cn/). Graphene oxide was purchased from XFNANO Co. Ltd. (Nanjing, China, http://www.xfnano.com). The deoxyribonuclease I (DNase I) were obtained from solarbio (Beijing, China, http://www.solarbio.com). The MUC1 (5′-FAM-CCCGTCTTCCAGACAAGAGTGCAGGG-3′) aptamer were synthesized by Sangon Biological Engineering Technology Co., Ltd. (Shanghai, China, http://www.sangon.com) and purified using high performance liquid chromatography. All of the reagents were diluted to the required concentration with working buffer (10 mM Tris, 100 mM NaNO_3_ pH 7.4) before usage. Healthy human serum, urine and saliva were provided by Affiliated Dongfeng Hospital, Hubei University of Medicine, and approved by Hospital's Ethics Committee. The other reagents employed were of analytical grade and used without further purification. Ultrapure water obtained from a Millipore water purification system (18.2 MΩ cm resistivity, Milli-Q Direct 8) was used in all runs.

### 2.2 Optimization of sensing conditions

Firstly, the 40 nM FAM labeled aptamer (probe) and a desired concentration of MUC1 was first mixed and kept at 37 °C for 15 min, followed by adding 15 μg mL^−1^ of GO. 5 min later, the reaction solution was added with 2 U mL^−1^ DNase I and incubated at 37 °C for 15 min, then the solution was diluted to 1 mL. Finally, the fluorescence of the mixture were carried out on a Hitachi F-4600 spectrophotometer (Hitachi Co. Ltd, Japan, http://www.hitachi.co.jp) equipped with a xenon lamp excitation source at room temperature. The excitation was set at 495 nm and the emission spectra were collected from 510 nm to 600 nm. The slits of the excitation and emission were both set at 5 nm. The fluorescence intensity at 519 nm was used to choose the optimal experimental conditions and evaluate the performance of the proposed sensing system. In control experiments, the measurement process was all the same with the above except the addition of MUC1. Unless otherwise noted, each fluorescence measurement was repeated three times, and the standard deviation was plotted as the error bar.

## 3. Results and discussion

### 3.1. Principle of design

**Fig. 1:**
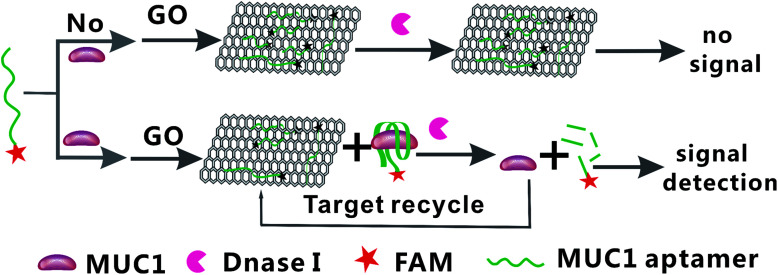
Schematic illustration of the GO-based fluorescent aptasensor assay detection of MUC1 by using DNase I-mediated target cyclic amplification.

In the present study, the principle of fluorescent aptasensor detection of MUC1 based on DNase I-aided target recycling signal amplification is illustrated in Fig. 1. In the absence of MUC1, the GO can strongly adsorb single-stranded nucleic acids, thus, the MUC1 probe is adsorbed onto the surface of GO *via* π–π stacking between DNA bases and GO, leading to the fluorescence is quenched, resulting in no fluorescence signal. Upon adding the MUC1, the probe sequences could be specific recognized by MUC1 to form probe/MUC1 complexes, leading to increase in the fluorescence intensity. Then, when the DNase I is added, all DNA sequences are degraded, resulting in the release of MUC1 and FAM fluorophores. The released MUC1 is recycled repeatedly, leading to the accumulation of free FAM fluorophores. The quantities of MUC1 can be achieved by fluorescence increment. Thus, the fluorescence signal is significantly amplified by DNase I-mediated target recycling process.

### 3.2. Feasibility analysis of the developed method for MUC1 detection

**Fig. 2:**
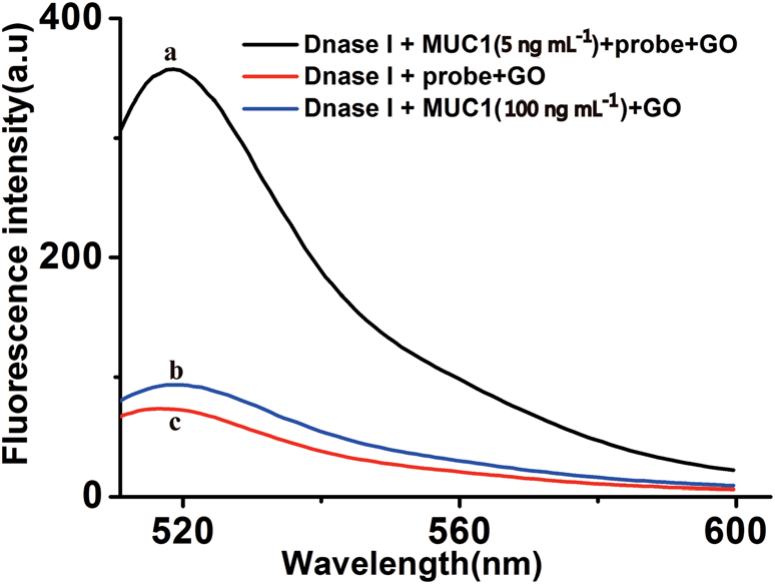
The feasibility analysis. (a) Dnase I + MUC1 (5 ng mL^−1^) + probe + GO; (b) Dnase I + MUC1 (100 ng mL^−1^) + GO; (c) Dnase I + probe + GO. The concentrations of Dnase I, GO and probe were 2 U mL^−1^, 15 μg mL^−1^ and 40 nM, respectively.

To further verify the feasibility of DNase I-aided target recycling signal amplification strategy, Fig. 2 shows the fluorescence emission spectra under different conditions. The fluorescence signal produced by mixture solution DNase I and GO (curve c) is relatively weak in the absence of MUC1, indicating that GO can protect the probe from digesting by DNase I, resulting in the no FAM fluorophores are produced. And then, when the MUC1 is added, the fluorescence intensity do not obviously increase (curve b) in the absence of probe, thus, the MUC1 cannot specifically bind to probe, lead to the no FAM fluorophores are produced.

However, upon adding MUC1 and probe, significant enhancement of the fluorescence intensity was observed (curve a), as a result of the specific binding of probe to MUC1, lead to the probe/MUC1 complex formation, and keep the FAM fluorophores away from GO. More importantly, the DNase I-aided target recycling results in the significant fluorescence amplification. Taking these results together, the feasibility of the proposed aptasensor for MUC1 detection by our design.

### 3.3. Optimization of reaction conditions

**Fig. 3:**
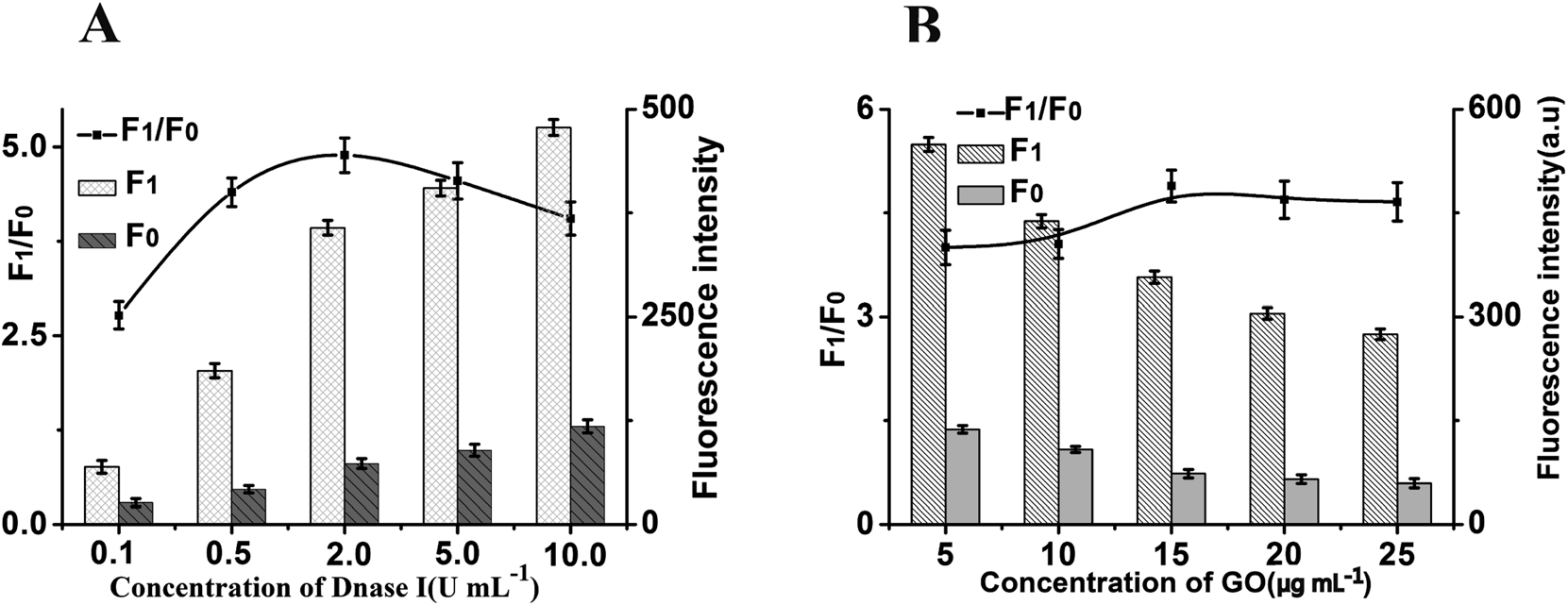
(A) The effect of Dnase I concentration on the fluorescence response of this method. (B) The effect of GO concentration on the fluorescence response of this method. *F*_0_: control experiments; *F*_1_: with 5 ng mL^−1^ of MUC1, and the concentrations of probe is 40 nM. The black lines represent the *F*_1_/*F*_0_ at different conditions, while the *F*_1_ and *F*_0_ were the fluorescence intensities in the presence and absence of MUC1, respectively. Error bars: SD, *n* = 3.

To achieve optimal sensing performance, several reaction conditions such as the concentration of DNase I, the concentration of GO, the enzyme digestion time and reaction temperature were optimized. While the *F*_1_ and *F*_0_ were the fluorescence intensities in the presence and absence of MUC1, respectively. The fluorescence intensity and the value of *F*_1_/*F*_0_ are selected to evaluate the effects of the reaction conditions on the sensing performance of the method. As shown in Fig. 3A, Maximum *F*_1_/*F*_0_ value is observed when the concentration of DNase I was 2 U mL^−1^, however, *F*_1_/*F*_0_ value decreases obviously along with the further increasing of DNase I concentration. Thus, a DNase I concentration of 2 U mL^−1^ was confirmed as the optimized concentration. At the same time, the concentration of GO is another important factor affecting fluorescence intensity. As depicted in Fig. 3B, with the increase in the concentration of GO, the fluorescence intensity initially increased and then gradually decrease, and maximum *F*_1_/*F*_0_ values is observed when the concentration of GO is 15 μg mL^−1^. Thus, 15 μg mL^−1^ GO are used in the subsequent experiments.

**Fig. 4:**
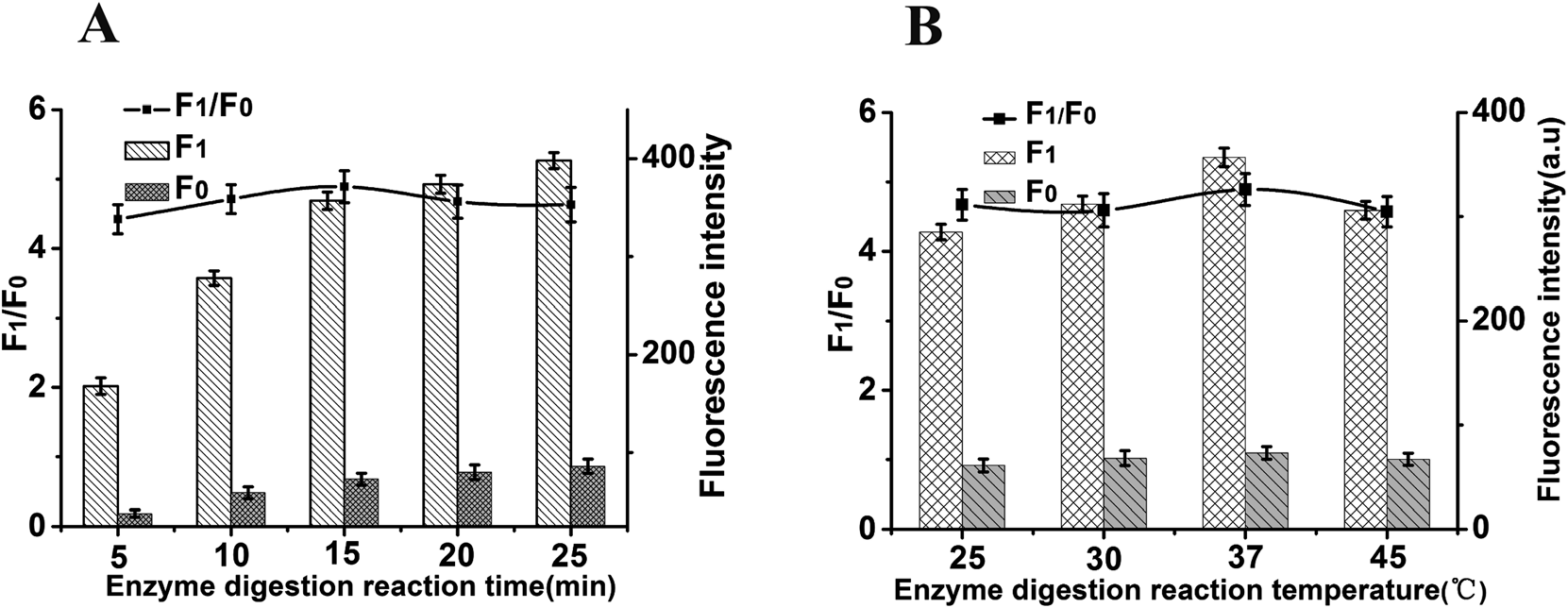
The effect of enzyme digestion reaction time and reaction temperature on the fluorescence intensity at the emission wavelength of 519 nm. The concentrations of Dnase I, MUC1, probe and GO were 2 U mL^−1^, 5 ng mL^−1^, 40 nM and 15 μg mL^−1^, respectively. Error bars: SD, *n* = 3.

In addition, the enzyme digestion time and reaction temperature are another important reaction condition affecting fluorescence intensity for this sensor. The fluorescence intensity and the value of *F*_1_/*F*_0_ change is related to the enzyme digestion reaction time. As shown in Fig. 4A, maximum *F*_1_/*F*_0_ values is observed when enzyme digestion time is 15 min. Thus, 15 min of enzyme digestion time was selected for the rest of the experiments. Fig. 4B shows that the enzyme digestion temperature could obviously affect the sensitivity; the *F*_1_/*F*_0_ value reached a maximum when enzyme digestion temperature was 37 °C and then decreased gradually. Therefore, 37 °C was confirmed as the optimized enzyme digestion temperature.

To obtain high sensitivity, the concentrations of aptamer were also investigated. As shown in Fig. S1,† the concentration of aptamer was selected in the following experiments; the *F*_1_/*F*_0_ value reached a maximum when the concentration of aptamer was 40 nM. Therefore, a concentration of 40 nM was selected as the optimal concentration of aptamer.

### 3.4. Sensitivity and specificity

**Fig. 5:**
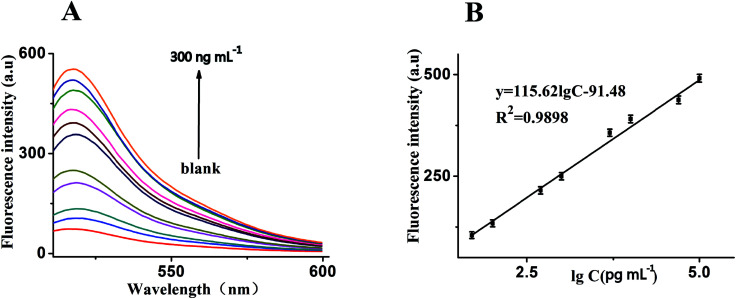
(A) Fluorescence emission spectra of the biosensor in the presence of MUC1 with different concentrations: from bottom to top: 0, 10 pg mL^−1^, 50 pg mL^−1^, 100 pg mL^−1^, 500 pg mL^−1^, 1 ng mL^−1^, 5 ng mL^−1^, 10 ng mL^−1^, 50 ng mL^−1^, 100 ng mL^−1^, 200 ng mL^−1^ and 300 ng mL^−1^, respectively. (B) The relationship curve of the fluorescence intensity as a function of MUC1 concentration. While shows the relationship between fluorescence intensity and MUC1 concentration, both experimental conditions: Dnase I, 2 U mL^−1^; MUC1, 5 ng mL^−1^; probe, 40 nM; GO, 15 μg mL^−1^ and the emission wavelength of 519 nm. Error bars: SD, *n* = 3.

**Fig. 6:**
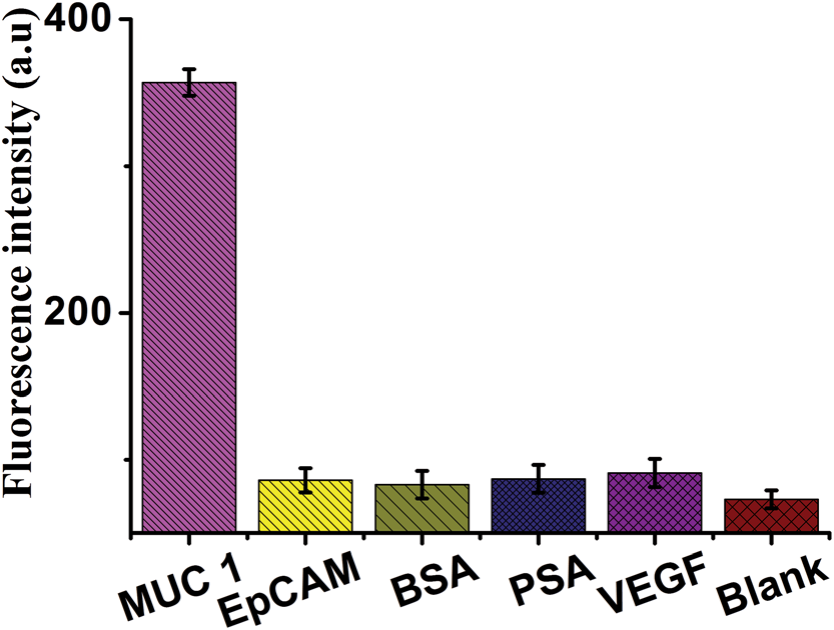
Fluorescence intensity (at the emission wavelength of 519 nm) of the sensor in the presence of MUC1 (5 ng mL^−1^), EpCAM (50 ng mL^−1^), BSA (50 ng mL^−1^), PSA (50 ng mL^−1^), VEGF (50 ng mL^−1^) and black, respectively. Error bars: SD, *n* = 3.

Under the optimal reaction conditions, the sensitivity of the sensor for detection of MUC1 is investigated. Fig. 5A shows the fluorescence emission spectra of the biosensor incubated in different concentrations of MUC1. We found that the fluorescence dramatically enhance with the increasing concentration of MUC1 from 0 to 300 ng mL^−1^. It illustrates a highly concentration dependence of the sensor for detection of MUC1. At the same time, Fig. 5B shows a good linear correlation between the fluorescence intensity and the concentration of MUC1 in the range from 50 pg mL^−1^ to 100 ng mL^−1^. The calibration plot of the linear equation is given as *y* = 115.62 lg *c* − 91.48 (*R*^2^ = 0.9898), where lg *c* is logarithm of MUC1 concentration and *y* is the fluorescence intensity. Furthermore, the detection limit is at 10 pg mL^−1^. In comparison, it is much lower than other sensors reported in literatures listed in Table 1. In addition, for the specificity study, adding different control proteins was investigated, including EpCAM (50 ng mL^−1^), BSA (50 ng mL^−1^), PSA (50 ng mL^−1^) and VEGF (50 ng mL^−1^). As shown in Fig. 6, in the presence of other control proteins (50 ng mL^−1^), the significant increase of fluorescence signal is observed in the presence of the MUC1 (5 ng mL^−1^), indicating that this proposed strategy exhibited good specificity for MUC1 detection.

**Table 1:** The comparison of designed method for the detection of MUC1 with other reported biosensors

Analytical method	Detection limit	Linear range	Ref.
Colorimetric biosensor	1.68 μg mL^−1^	15–100 μg mL^−1^	8
Electrochemical	9.3 fg mL^−1^	0.94 fg mL^−1^ to 9.38 μg mL^−1^	13
Electrochemical	21 ng mL^−1^	0–84 ng mL^−1^	14
Electrochemical	1.2 μg mL^−1^	0.65–110 ng mL^−1^	15
Electrochemical	1.2 ng mL^−1^	3 ng mL^−1^ to 3 g mL^−1^	16
Electrochemistry	0.62 ng mL^−1^	1–12 ng mL^−1^	9
Electrochemistry	0.15 μg mL^−1^	0.3–50 μg mL^−1^	10
Electrochemistry	0.66 μg mL^−1^	2.64–105.99 μg mL^−1^	11
Photoelectrochemical	0.16 μg mL^−1^	0.6–20 μg mL^−1^	12
Electrochemiluminescence	0.62 fg mL^−1^	1 fg mL^−1^ to 1 ng mL^−1^	17
Electrochemiluminescence	3.33 fg mL^−1^	10 fg mL^−1^ to 10 ng mL^−1^	19
Electrochemiluminescence	2.8 fg mL^−1^	10 fg mL^−1^ to 30 ng mL^−1^	18
Fluorescent biosensor	0.03 μg mL^−1^	0.03–15 μg mL^−1^	20
Fluorescent biosensor	1 ng mL^−1^	3 ng mL^−1^ to 1.5 μg mL^−1^	21
Fluorescent biosensor	5.13 μg mL^−1^	6–241.2 μg mL^−1^	22
Fluorescent biosensor	8.4 μg mL^−1^	12 μg mL^−1^ to 3 mg mL^−1^	23
Fluorescent biosensor	10 pg mL^−1^	50 pg mL^−1^ to 100 ng mL^−1^	**This method**

### 3.5. Determination of MUC1 in real samples

**Fig. 7:**
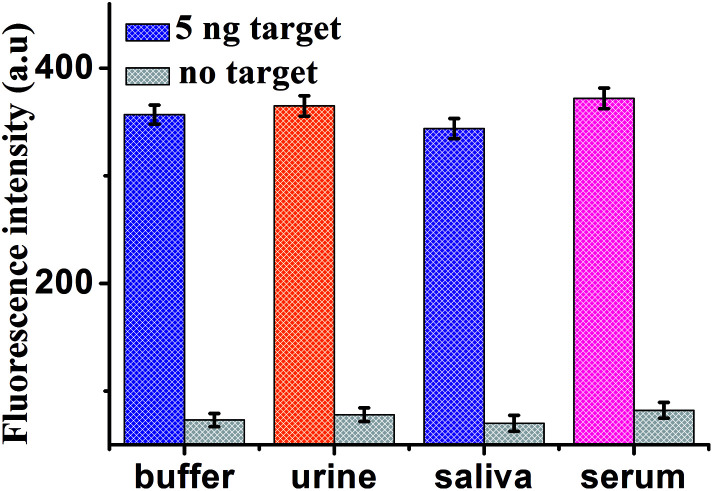
Fluorescence intensity of the sensor for detection of MUC1 in buffer and blank biological samples (human urine, saliva and serum, respectively). Error bars: SD, *n* = 3.

To further verify the potential applicability of this present strategy in biological samples, the detection of MUC1 in biological samples by spiking MUC1 to human urine, saliva and serum diluted to 10% with buffer solution with the final concentration of 5 ng mL^−1^ were performed. As shown in Fig. 7, a significant increase in various biological samples is observed, compared with no spiking biological samples. These results clearly demonstrate that this sensor can be a potential analytical method to detect MUC1 in real samples sensitively. The recoveries for the various concentrations of spiked MUC1 in human serum were in the range of 108.4–111.2%, with the relative standard deviations (RSDs) of 7.9%, 9.8% and 8.9% at 0.1, 5.0 and 50.0 ng mL^−1^ of MUC1, respectively. This indicates an acceptable precision and reproducibility of the present approach for detecting MUC1 in real samples (*n* = 3) (Table 2).

**Table 2:** Recovery of MUC1 spiked in human serum samples

Serum sample	Added MUC1/ng	MUC1 found/nM	Recovery (100%)	RSD (%)
1	0	0	—	—
2	0.10	0.11^a^	110.0	7.9
3	5.0	5.42^a^	108.4	9.8
4	50.0	55.6^a^	111.2	8.9

a The average value of three measurements.

## 4. Conclusions

In summary, we have successfully developed an amplified GO-based fluorescence aptasensor for detecting MUC1. In this design, because of the deoxyribonuclease I-aided target recycling signal amplification, and based on GO efficient fluorescence quencher, selectivity and sensitivity for MUC1 were achieved. As to MUC1, this sensor can detect MUC1 with a linear range from 50 pg mL^−1^ to 100 ng mL^−1^, and the detection limit was low to 10 pg mL^−1^. In addition, the fluorescence signals are also obtained when this sensor is used for biological samples. More importantly, this biosensor achieved a high sensitivity by DNase I-aided target recycling and accumulated plenty of FAM fluorophores. Thereby, this design provides an efficient platform for MUC1 detection, may be a potential method for tumor diagnosis, bioanalysis, and clinical analysis.

## Conflicts of interest

There are no conflicts to declare.

## Supplementary Material

RA-008-C8RA06498A-s001
