# Supplementary material for: Ultrasensitive fluorescent aptasensor for MUC1 detection based on deoxyribonuclease I-aided target recycling signal amplification
Source: RSC Adv. 2018 Sep 14;8(56):32009–15. doi: 10.1039/c8ra06498a (PMC9085725; doi:10.1039/c8ra06498a)
Supplement: RA-008-C8RA06498A-s001 [file RA-008-C8RA06498A-s001.pdf]

Electronic Supplementary Material (ESI) for RSC Advances.
This journal is © The Royal Society of Chemistry 2018

Optimization of Aptamer

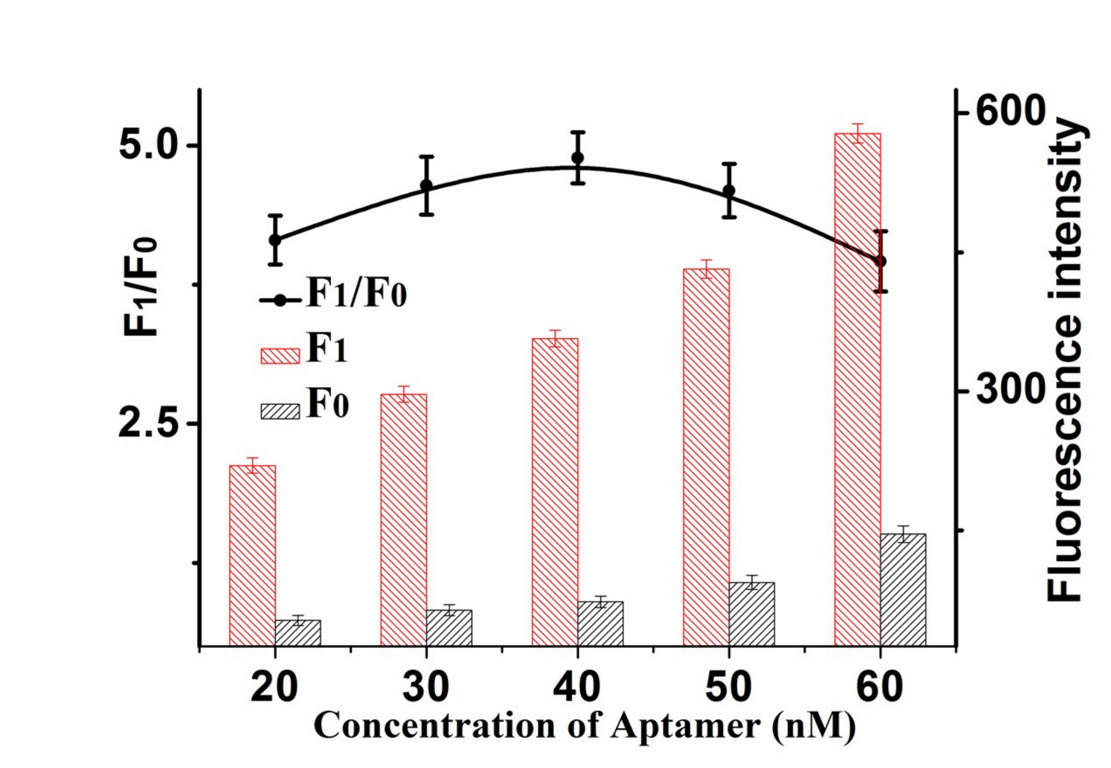

Fig.S1. The effect of Aptamer on the fluorescence intensity at the emission wavelength of 519 nm. The concentrations of Dnase I, MUC1 and GO were 2 U·mL^{-1}, 5 ng·mL^{-1} and 15 μg·mL^{-1}, respectively. Error bars: SD, n=3.
